# Identification of the Signature Associated With m^6^A RNA Methylation Regulators and m^6^A-Related Genes and Construction of the Risk Score for Prognostication in Early-Stage Lung Adenocarcinoma

**DOI:** 10.3389/fgene.2021.656114

**Published:** 2021-06-11

**Authors:** Bingzhou Guo, Hongliang Zhang, Jinliang Wang, Rilige Wu, Junyan Zhang, Qiqin Zhang, Lu Xu, Ming Shen, Zhibo Zhang, Fangyan Gu, Weiliang Zeng, Xiaodong Jia, Chengliang Yin

**Affiliations:** ^1^School of Mathematical Sciences, Harbin Normal University, Harbin, China; ^2^Department of Emergency, The First Medical Center of Chinese PLA General Hospital, Beijing, China; ^3^Department of Oncology, The Second Medical Center of Chinese PLA General Hospital, Beijing, China; ^4^National Engineering Laboratory for Medical Big Data Application Technology, Chinese PLA General Hospital, Beijing, China; ^5^Medical Big Data Research Center, Medical Innovation Research Division of Chinese PLA General Hospital, Beijing, China; ^6^Department of Orthopedics, Weifang Traditional Chinese Hospital, Weifang, China; ^7^Laboratory of Translational Medicine, Medical Innovation Research Division of Chinese PLA General Hospital, Beijing, China; ^8^The 78th Group Army Hospital of Chinese PLA, Mudanjiang, China; ^9^Clinical Biobank Center, Medical Innovation Research Division of Chinese PLA General Hospital, Beijing, China; ^10^Department of Liver Disease, Fifth Medical Center of Chinese PLA General Hospital, Beijing, China; ^11^Faculty of Medicine, Macau University of Science and Technology, Macau, China

**Keywords:** lung adenocarcinoma, m^6^A, prognostic signature, m^6^A-related genes, RNA methylation regulators

## Abstract

**Background:**

N6-methyladenosine (m^6^A) RNA modification is vital for cancers because methylation can alter gene expression and even affect some functional modification. Our study aimed to analyze m^6^A RNA methylation regulators and m^6^A-related genes to understand the prognosis of early lung adenocarcinoma.

**Methods:**

The relevant datasets were utilized to analyze 21 m^6^A RNA methylation regulators and 5,486 m^6^A-related genes in m^6^Avar. Univariate Cox regression analysis, random survival forest analysis, Kaplan–Meier analysis, Chi-square analysis, and multivariate cox analysis were carried out on the datasets, and a risk prognostic model based on three feature genes was constructed.

**Results:**

Respectively, we treated GSE31210 (*n* = 226) as the training set, GSE50081 (*n* = 128) and TCGA data (*n* = 400) as the test set. By performing univariable cox regression analysis and random survival forest algorithm in the training group, 218 genes were significant and three prognosis-related genes (*ZCRB1*, *ADH1C*, and *YTHDC2*) were screened out, which could divide LUAD patients into low and high-risk group (*P* < 0.0001). The predictive efficacy of the model was confirmed in the test group GSE50081 (*P* = 0.0018) and the TCGA datasets (*P* = 0.014). Multivariable cox manifested that the three-gene signature was an independent risk factor in LUAD. Furthermore, genes in the signature were also externally validated using the online database. Moreover, YTHDC2 was the important gene in the risk score model and played a vital role in readers of m^6^A methylation.

**Conclusion:**

The findings of this study suggested that associated with m^6^A RNA methylation regulators and m^6^A-related genes, the three-gene signature was a reliable prognostic indicator for LUAD patients, indicating a clinical application prospect to serve as a potential therapeutic target.

## Introduction

Lung adenocarcinoma (LUAD) is a type of non-small cell cancer. In the 2018 Global Cancer Report, lung cancer ranked top 1 with the highest incidence and mortality among all cancers (Bray et al., 2018).

N6-methyladenosine (m^6^A) RNA methylation is the most abundant epigenetic modification in eukaryotic mRNA. M^6^A methylation regulators of each modified RNA require a writer to place, an eraser to erase, and a reader to read. Based on these proteins, m^6^A affected RNA splicing (He et al., 2019), translation, and RNA stability (Wang et al., 2014; He et al., 2019). Evidence is now mounting that m^6^A methylation underlies the progression of tumors and affects specific biological processes through non-coding RNA modification (Xiao et al., 2019). Moreover, the over-expression of YTHDF1 in the reader might affect the prognosis of ovarian cancer patients (Liu et al., 2020). In the writer family, high expression of METTL3 promoted the proliferation of bladder cancer (Cheng et al., 2019) and led to a poor prognosis (Han et al., 2019). Over-expression knockdown of ALKBH5 could effectively reduce cell proliferation in pancreatic cancer in erasers family (Tang et al., 2020). Meanwhile, m^6^A has many functions in cancer (He et al., 2019; Ma et al., 2019; Ma and Ji, 2020), such as reduced m^6^A has a relationship with phenotypes of gastric cancer (Zhang et al., 2019), KIAA1429 is associated with prognosis of liver cancer (Lan et al., 2019), and FTO could facilitate the development of breast cancer (Niu et al., 2019). However, to our knowledge, there are few studies related to m^6^A methylation in early LUAD, and this may be a novel treatment strategy for patients with early LUAD.

In this study, GEO and TCGA data were used to explore the influence of m^6^A methylation genes and their regulated genes on the prognosis of early lung adenocarcinoma. The signature was conducted for identifying new therapeutic biomarkers and treatment strategy development.

## Materials and Methods

### Expression Data

Data was downloaded from Gene Expression Omnibus (GEO) and The Cancer Genome Atlas (TCGA) public databases. GSE31210 (*n* = 226) was used as the training set, GSE50081 (*n* = 128) as the validation set 1, and TCGA (*n* = 400) data as the validation set 2. Three independent datasets were used for model construction and model verification. Each independent dataset included the clinical characteristics: survival status, survival time, age, sex, and clinical TNM stage. In GEO data, TNM clinical stage was divided into stages I and II, which were shown in Table 1. Besides, the GPL570 chip platform was re-annotated by the probe to get the final expression profile of the GEO data (Fan et al., 2018). Only mRNA probes were selected, and 8,597 mRNA expression profiles were obtained.

**TABLE 1:** Clinical information of the Gene Expression Omnibus (GEO) and The Cancer Genome Atlas (TCGA) datasets.

**Characteristic**	**GSE31210**	**GSE50081**	**TCGA**
**Age (years)**			
>61	104	104	251
≤61	122	24	149
**Sex**			
Female	121	63	217
Male	105	65	183
**Vital status**			
Alive	191	76	278
Dead	35	52	122
**Pathological stage**			
Stage I	168	92	280
Stage II	58	36	120

### Selection of m^6^A RNA Methylation Regulatory Factors and m^6^A-Related Genes

We collected 21 m^6^A methylated genes through literature investigation (Supplementary Table 1) (Zhang et al., 2020). We found that among these 21 genes, 14 genes were expressed in the training set (GSE31210). In LUAD, a total of 5,486 m^6^A-regulated genes were downloaded from the m^6^Avar database^1^ (Zheng et al., 2018). Among the 5,507 genes, 2,615 genes were expressed in GSE31210.

Discovery of the m^6^A RNA Methylation Regulators and m^6^A-Related Genes and Establishment of the m^6^A Methylation Risk Score Model.

We obtained prognostic-related gene sets through survival analysis [univariate cox and Kaplan–Meier (KM)] and receiver operating characteristic (ROC) curve. In the training set GSE31210, we used the random survival forest (RSF) (Ishwaran et al., 2008) to establish a prognostic model related to patient overall survival (OS). Methods of analyzing survival data were often parametric, nonlinear effects of variables, and modeled by expanding matrix for specialized functions. Identifying multiple variable interactions was also problematic. These difficulties could be effectively solved using RSF (Ishwaran et al., 2008). Its basic formula is:

$$\mathrm{RSF}=\sum_{i=1}^{N}\text{Expi}\times \text{Coefi}$$

The meanings of the parameters in this formula are: RS is the risk score, *N* is the number of genes, Exp is the expression amount of genes in the data, and Coef is the coefficient of cox analysis for the genes resulting from the random survival forest. We used gene combinations to select the largest AUC to construct a prognostic model. Based on the median of the risk scores, the data were divided into two groups: the high-risk group and the low-risk group. The KM curve was used to compare the difference between the high and low-risk groups. In the three datasets, using the median score divided two groups.

### Estimation of Outcome Signature for Patients’ Prognosis and Its Relationship With Clinical Characteristics

To assess the characteristics of the patients’ prognosis and its relationship with clinical features, we used chi-square analysis to judge the correlation between the model and clinical data. KM survival curve and log-rank test were used to describe the relationship between the model and OS. Furthermore, multivariate Cox regression analysis was used to study whether the clinical data (age, gender, and pathological stage) were related to OS in the training set and validation set. We used univariate Cox analysis to judge whether the clinical information had prognostic value.

### External Validation of the Genes in the Gene Signature

Furthermore, four online tools were used to verify the gene expression levels (Oncomine database^2^; TIMER database^3^, and GEPIA database, Gene Expression Profiling Interactive Analysis^4^) and protein levels [The Human Protein Atlas (HPA) database^5^ ] in the model. Meanwhile, online public databases (The cBioPortal for Cancer Genomics^6^) were used to analyze and understand the gene influence on early treatment of LUAD.

### Function Notes and Protein–Protein Interaction

The R package ‘‘clusterProfiler’’ was used to annotate the function and select the statistically significant pathways. The relationship between proteins was analyzed by using the online website STRING^7^ (Szklarczyk et al., 2019). Cytoscape was used to visualize the network. Then the main networks were chosen by a degree of the gene in the net analysis.

### Statistical Analysis

In three independent datasets, all KM and cox analyses were performed using the R package “survival”. Cox analysis was used to select prognostic genes and test models. “ROC” and “TimeROC” were available to verify the feasibility of the model. Functional annotations were made using the R package “Clusterprofiler.” All of our analyses (besides online website analysis) were performed in the R language. R packages were used as follows: “pROC,” “TimeROC,” “survival,” “clusterProfiler’,” and “randomForestSRC.” The *P* values of the above analyses were all <0.05 as statistically significant.

## Results

### Patient Population

All 226, 128, and 400 patients diagnosed with LUAD were collected from the GEO (GSE31210 and GSE50081) and TCGA database, respectively. A total of 2,615 m^6^A-related genes out of the genes expressed were identified in the GSE31210 dataset. In Table 1, the median age of the enrolled samples was 61 years. The ratio of male vs. female was 1.15:1, with 191 live cases and 35 death cases. The longest survival was 10 years. Each sample data was only distributed in stages I–II of LUAD. The study workflow is demonstrated in Supplementary Figure 1.

### Construction of the Risk Score Model, the m^6^A RNA Methylation Regulators, and m^6^A-Related Genes Risk Score

**FIGURE 1:**
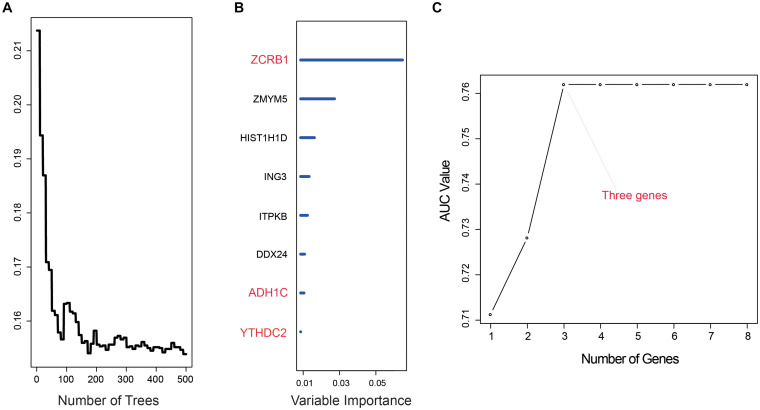
Random survival forest analysis. **(A,B)** random survival forests variable hunting analysis reveals the error rate for the data as a function of trees and uses the associated score to filter N6-methyladenosine (m^6^A) RNA methylation regulators and m^6^A-related genes. **(C)** Receiver operating characteristic (ROC) for selected prognostic signature from all 255 signatures.

After we used univariate cox analysis and ROC curve, 218 prognostic-related genes were selected, and the screening criteria were *P* < 0.01 and AUC > 0.6 in Supplementary Table 2. Furthermore, gene screening was performed by the importance scores of the random survival forest analysis. We permuted and combined the eight genes selected from the random survival forest, obtaining 2^8^ – 1 = 255 prediction models (Figures 1A,B). The 255 models were evaluated by AUC, and the optimal predictive ability was found in the combination of three genes, ZCRB1, ADH1C, and YTHDC2. As a prediction model, the AUC of the three-gene model was 0.762 (Figure 1C). The risk score of the model was RSF = (1.725151 × ZCRB1) + (−1.964326 × ADH1C) + (−2.015378 × YTHDC2). Each gene name represented its expression level in a certain sample.

**FIGURE 2:**
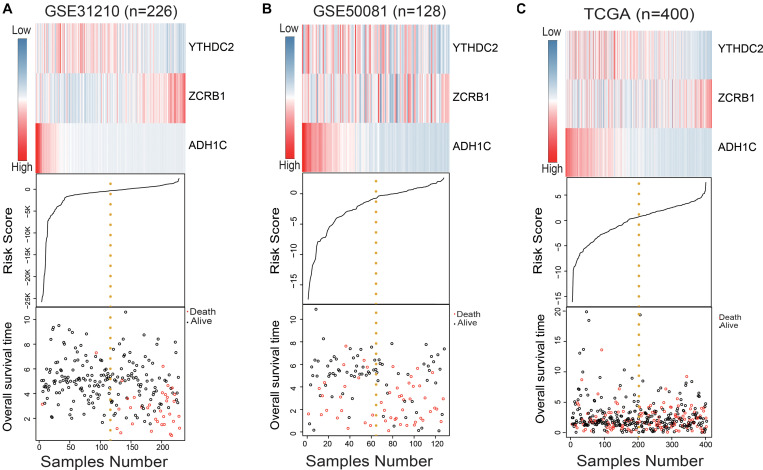
Evaluation of the risk predictive model in the training set and test set. **(A–C)** The distribution of m^6^A RNA methylation regulators and m^6^A-related gene expression level, patients’ survival status, and risk score between high- and low-risk group.

We used the RSF formula to calculate the risk score of each sample and plotted the heat map of the three genes (Figures 2A–C), finding that in the high-risk group, ADH1C and YTHDC2 basically had low expression, while ZCRB1 obviously had high expression. This was particularly evident in the training group (GSE31210) (Figure 2A).

The results made it clear that ADH1C had high expression in the low-risk group as a protection factor by cox analysis (Table 2).

**TABLE 2:** Prognosis of the three genes in the signature.

**ENSEMBL ID**	**Symbol ID**	**Gene name**	**Coef**	***P*-value**	**Prognostic indicator**
ENSG00000047188	YTHDC2	YTH domain containing 2	−2.02	0.00	low
ENSG00000139168	ZCRB1	zinc finger CCHC-type and RNA binding motif containing 1	1.73	0.00	high
ENSG00000248144	ADH1C	alcohol dehydrogenase 1C (class I), gamma polypeptide	−1.96	0.00	low

### The Validation of Performance in Predicting Overall Survival

**FIGURE 3:**
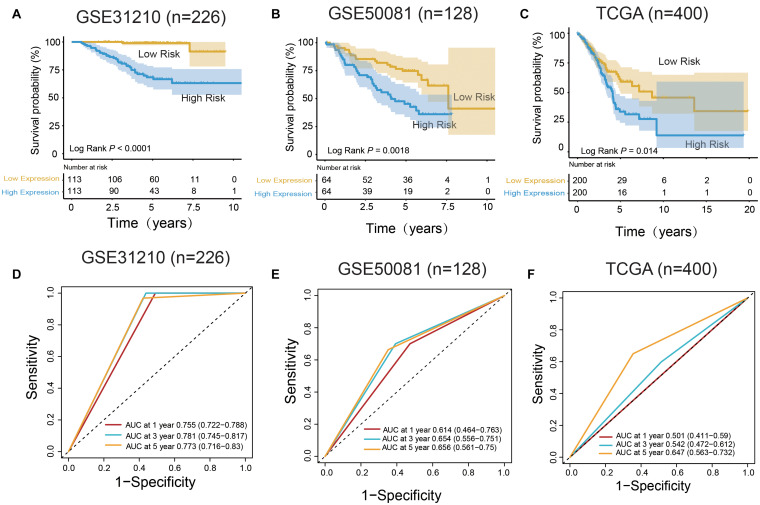
m^6^A RNA methylation regulators and m^6^A-related genes signature predict overall survival of patients of LUAD. **(A–C)** Kaplan–Meier survival curves classify patients into high- and low-risk groups by the m^6^A RNA methylation regulators and m^6^A-related genes signature in the training dataset (GSE31210), and test dataset (GSE50081 and TCGA). *P* values were calculated by log-rank test. **(D–F)** m^6^A RNA methylation regulators and m^6^A-related genes signatures were used for predicting survival in 1, 3, and 5 years by TimeROC analysis.

In the training set, the median risk score divided all patients into two groups: high-risk group (*n* = 113) and low-risk group (*n* = 113) (Figure 3A). The KM survival curve and log rank test showed that our model had an excellent predictive power. In the validation set, the median risk score was also used to divide the patients into two groups in GSE50081 (*n* = 128), and there were 64 patients in the high-risk group and 64 patients in the low-risk group (Figure 3B). The KM survival curve showed that there was a significant difference between the high-risk group and low-risk group (Log rank *P* = 0.0018). Grouped by median risk score in TCGA (*n* = 400), there were 200 patients in the high-risk group and 200 samples in the low-risk group, with log rank *P* = 0.014 (Figure 3C).

In the training group (GSE31210), the 5-years survival rate was 53.10% in the low-risk group and 38.05% in the high-risk group (Figure 3A). Additionally, the overall survival rate was 45.58%, indicating that the risk score could differentiate the data correctly. Survival was significantly improved in the two independent validation data (GSE50081 and TCGA). Moreover, in GSE50081, the low-risk group was 56.25% and the high-risk group was 29.69% (Figure 3B). The overall survival rate was 42.97% and the grouping label was also evident. Meanwhile, in TCGA, we selected a sample data of TNM stage (I+II) (a total of 400 cases) (Figure 3C). Five-years survival rate was calculated in the high- and low-risk groups, and the rates were 14.5 and 8%, respectively. The overall 5-years survival rate was 11.25% in both low- and high-risk groups, and the survival rate in the low-risk group had markedly improved. Using time ROC in 5-years survival circumstances, we found that the label had an excellent prediction effect (Figures 3D–F). In GSE31210 and GSE50081, the AUC was 0.773 and 0.656, respectively, and the AUC was 0.647 in the TCGA.

### The Relationship Between the Signature and Clinical Characteristics

The association was demonstrated between the model and clinical information through the chi-square test in Table 3. There was a significant relationship between the pathological stage and the model (*P* < 0.05) in the GEO independent dataset rather than the TCGA dataset. Besides, there were 401 females in 754 cases, accounting for 53.18% of the total. A multivariate Cox test was utilized to determine if the signature had an independent prognostic value as a factor. The results in Table 4 showed that the signature was a risk factor, and it was statistically significant (high- vs. low-risk, GSE31210, HR = 16.24, 95% CI 3.85–68.58, *P* < 0.001, *n* = 226; GSE50081, HR = 2.23, 95% CI 1.24–4.02, *P* = 0.008, *n* = 128; TCGA, HR = 1.50, 95% CI 1.03–2.18, *P* = 0.036, *n* = 400). Univariate Cox also indicated that the signature was a risk factor.

**TABLE 3:** Clinical information and signature Chi-square table.

**Variables**	**Status**	**low**	**high**	***P***
**GSE31210 dataset (*n* = 226)**				
Age				0.89
	≤61	62	60	
	>61	51	53	
Gender				0.18
	Female	66	55	
	Male	47	58	
Pathological stage				0.00
	I	97	71	
	II	16	42	
**GSE50081 dataset (*n* = 128)**				
Age				0.82
	≤61	11	13	
	>61	53	51	
Gender				1.00
	Female	31	32	
	Male	33	32	
Pathological stage				0.03
	I	52	40	
	II	12	24	
**TCGA dataset (*n* = 400)**				
Age				0.00
	≤61	60	89	
	>61	140	111	
Gender				0.69
	Female	111	106	
	Male	89	94	
Pathological stage				0.10
	I	148	132	
	II	52	68	

**TABLE 4:** Univariable and multivariable Cox regression analysis of the signature and clinical information with lung adenocarcinoma (LUAD) survival.

		**Univariable cox**				**Multivariable cox**			
**Variables**		**HR**	**95% CI of HR**		***P***	**HR**	**95% CI of HR**		***P***
			**right**	**left**			**right**	**left**	
**GSE31210 (*n* = 226)**									
Age	>61 vs. ≤61	1.43	0.73	2.78	0.29	1.49	0.76	2.92	0.24
Sex	Male vs. female	1.52	0.78	2.96	0.22	1.03	0.51	2.08	0.92
Pathological stage	II vs. I	4.23	2.17	8.24	0.00	2.73	1.35	5.50	0.00
Signature	High risk vs. low risk	20.48	4.91	85.43	0.00	16.24	3.85	68.58	0.00
**GSE50081 (*n* = 128)**									
Age	>61 vs. ≤61	2.09	0.89	4.89	0.09	2.04	0.86	4.80	0.10
Sex	Male vs. female	1.35	0.78	2.34	0.29	1.51	0.86	2.64	0.15
Pathological stage	II vs I,	2.53	1.45	4.44	0.00	2.09	1.17	3.73	0.01
Signature	High risk vs. low risk	2.40	1.36	4.23	0.00	2.23	1.24	4.02	0.01
**TCGA (*n* = 400)**									
Age	>61 vs. ≤61	1.20	0.83	1.75	0.33	1.34	0.91	1.96	0.14
Sex	Male vs. female	1.03	0.72	1.47	0.87	1.03	0.72	1.48	0.88
Pathological stage	II vs. I	2.49	1.73	3.57	0.00	2.40	1.66	3.45	0.00
Signature	High risk vs. low risk	1.57	1.09	2.26	0.01	1.50	1.03	2.18	0.04

*CI, confidence interval; HR, hazard ratio.*

### Functional Annotation and Protein–Protein Interaction

**FIGURE 4:**
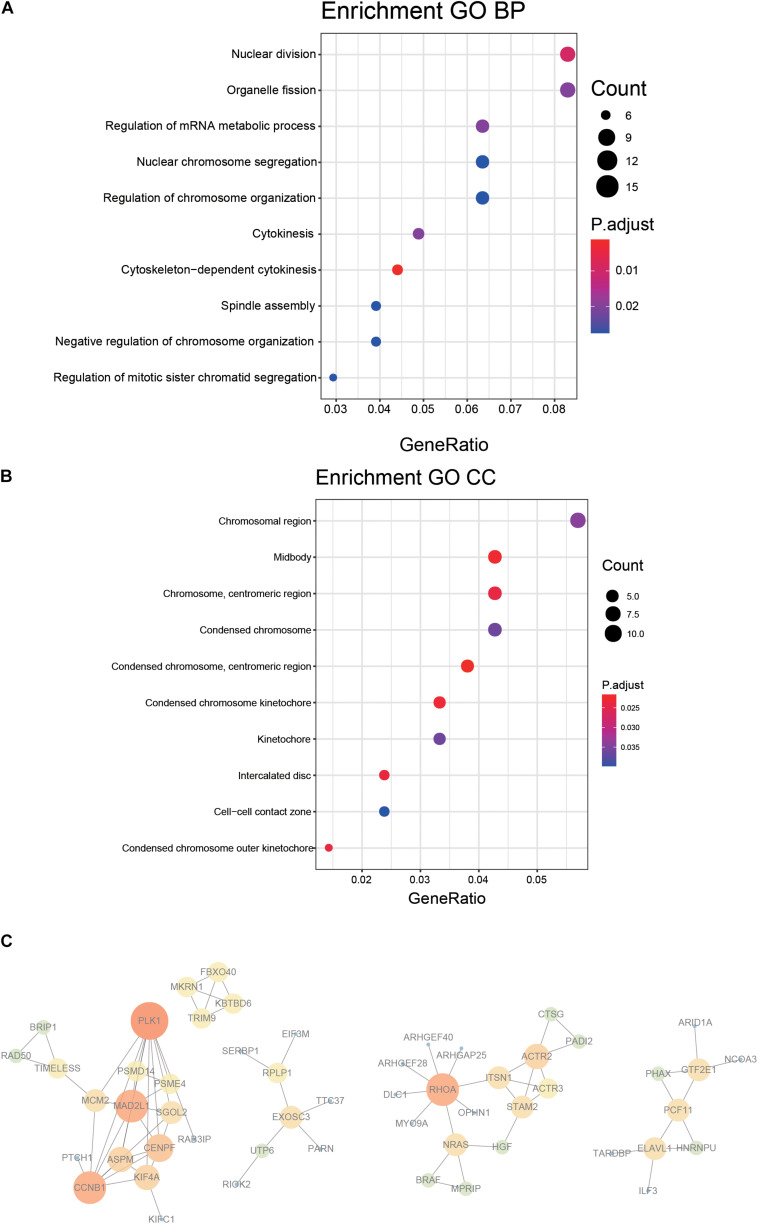
Functional annotation and protein–protein interaction for the genes with significant prognosis. **(A,B)** Function prediction (BP, biological process; CC, cellular component). **(C)** Protein–protein interaction.

A 218 gene set, obtained from survival analysis and AUC analysis, was used for functional annotation and PPI network analysis. Among the 218 genes, including m^6^A RNA methylation regulatory factors, ElAVL1, METTL14, and YTHDC2 were significantly associated with OS in LUAD. Top 10 biological processes (BPs) and cellular components (CCs) were selected by functional annotation of 218 genes, among which several results of BPs were related to division (nuclear division, organelle fission) and regulation (regulation of mRNA metabolic process and regulation of chromosome organization). The primary outcome of CCs was linked to the chromosome (Figures 4A,B).

PPI network was constructed by STRING, generated and visualized in Cytoscape. The combined score of the PPI criteria was >0.9. The PPI network had 88 relationships, and some genes were removed that were not part of the network (Figure 4C). Many key genes were observed in the network, such as PLK1, CCNB1, MAD2L1, RHOA, and ACTR2.

### External Validation Using Online Database About Genes in the Signature

**FIGURE 5:**
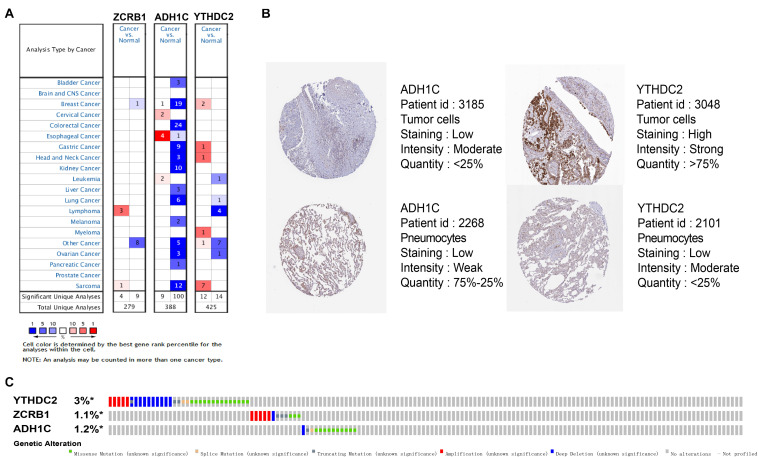
Expression and genetic alterations of the three predictive genes. **(A)** The expression profiles of the three genes in the Oncomine database. **(B)** The representative protein expression of the three genes in LUAD and normal lung tissue in the Human Protein Atlas database. Data of ZCRB1 were not found in the database. **(C)** Genetic alterations of the three genes in LUAD in the cBioportal for Cancer Genomics.

**FIGURE 6:**
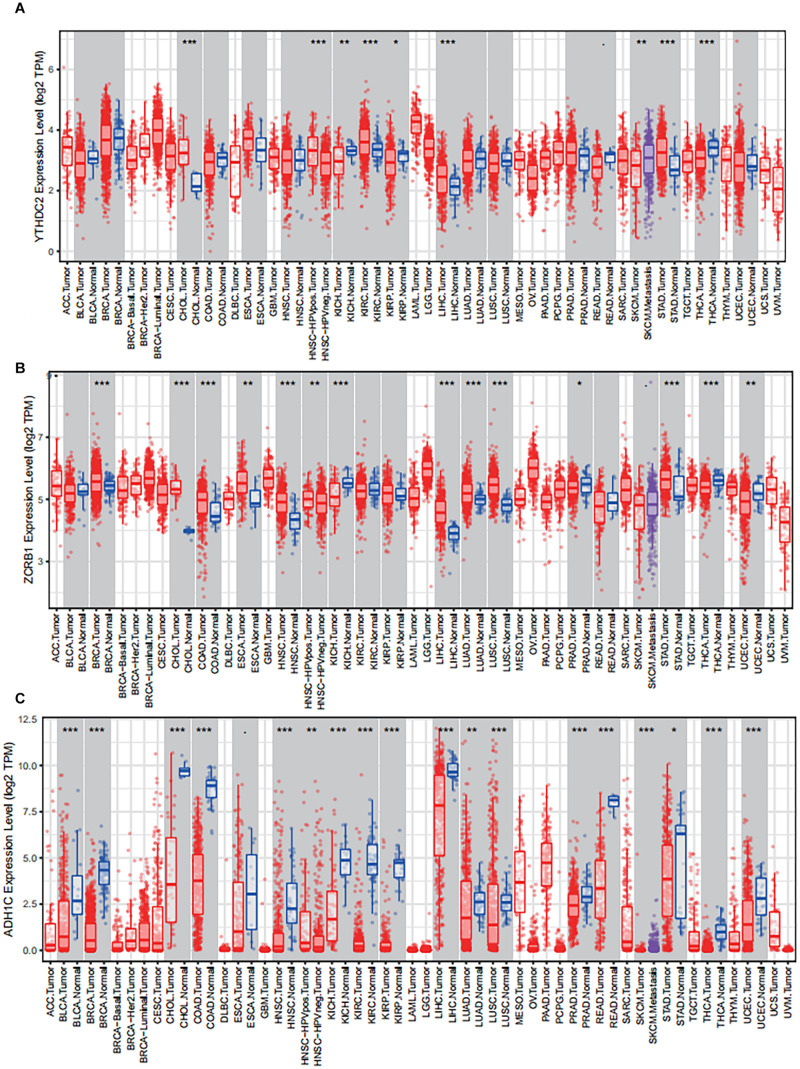
The expression of the three predictive genes in cancers via Tumor IMmune Estimation Resource (TIMER, https://cistrome.shinyapps.io/timer/). **(A)** YTHDC2 expression level in tumor tissues vs normal tissues. **(B)** ZCRB1 expression level in tumor tissues vs normal tissues. **(C)** ADH1C expression level in tumor tissues vs normal tissues. ACC, adrenocortical carcinoma; BLCA, bladder carcinoma; BRCA, breast carcinoma; CESC, cervical squamous cell carcinoma and endocervical adenocarcinoma; CHOL, cholangio carcinoma; COAD, colon adenocarcinoma; DLBC, lymphoid neoplasm diffuse large B-cell lymphoma; ESCA, esophageal carcinoma; GBM, glioblastoma multiforme; HNSC, head and neck squamous cell carcinoma; KICH, kidney chromophobe; KIRC, kidney renal clear cell carcinoma; KIRP, kidney renal papillary cell carcinoma; LAML, acute myeloid leukemia; LGG, brain lower grade glioma; LIHC, liver hepatocellular carcinoma; LUAD, lung adenocarcinoma; LUAC, lung squamous cell carcinoma; MESO, mesothelioma; OV, ovarian serous cystadenocarcinoma; PAAD, pancreatic adenocarcinoma; PCPG, pheochromocytoma and paraganglioma; PRAD, prostate adenocarcinoma; READ, rectum adenocarcinoma; SARC, sarcoma; SKCM, skin cutaneous melanoma; STAD, stomach adenocarcinoma; TGCT, testicular germ cell tumors; THCA, thyroid carcinoma; THYM, thymoma; UCEC, uterine corpus endometrial carcinoma; UCS, uterine carcinosarcoma; UVM, uveal melanoma.

The results of external validation data were consistent with our results. In LUAD, two genes YTHDC2 and ADH1C were lowly expressed in the three sets of independent data (Figure 5A), which was almost the same in both the TIMER database (Figure 6) and the GEPIA database (Supplementary Figure 2). Interestingly, the aberrant expression of the three genes were frequently observed in various cancers and showed some tissue-dependent patterns. For example, ZCRB1 was overexpressed in lymphoma, and ADH1C in cervical cancer and esophageal cancer, and YTHDC2 in breast cancer, gastric cancer, head and neck cancer, myeloma, and sarcoma (Figure 5A).

**FIGURE 7:**
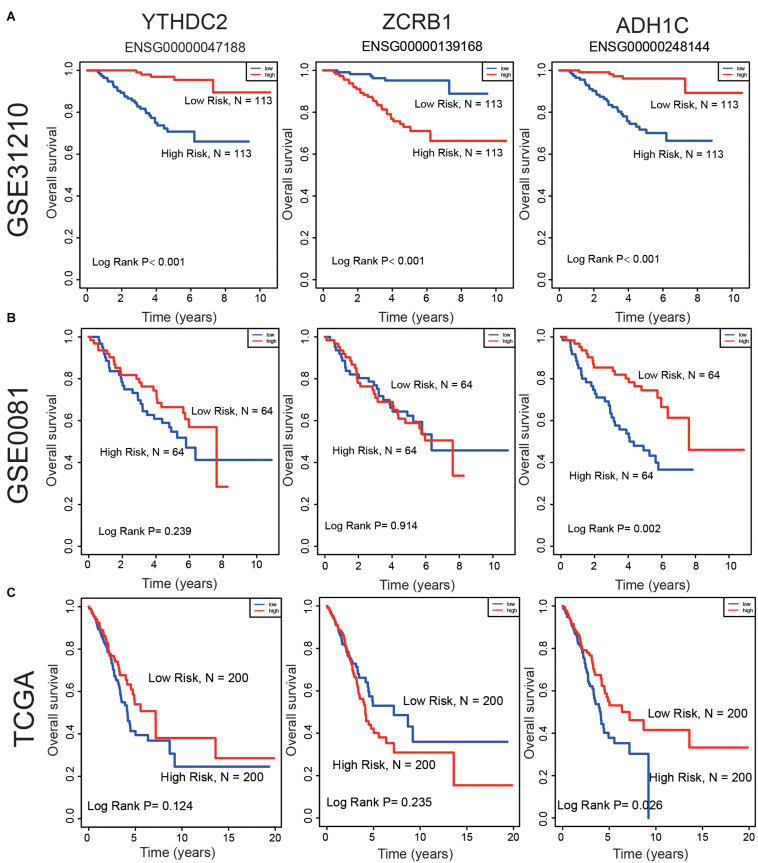
Compare the low and high expression of the three predictive genes in overall survival in **(A)** GSE31210 dataset, **(B)** GSE50081 dataset, and **(C)** TCGA dataset.

Survival analyses for each gene in the signature (ZCRB1, ADH1C, and YTHDC2) were performed in the cohorts of GSE31210, GSE50081, and TCGA datasets (Figure 7). ZCRB1 low-expression patient group displayed more OS than ZCRB1 high-expression patient group in GSE31210. While, ADH1C and YTHDC2 high-expression patient group displayed more OS than low-expression patient group not only in GSE31210 but also in GSE50081 and TCGA dataset.

We then reviewed the proteomic data and found YTHDC2 protein was reported significantly underexpressed in non-small cell lung cancer (Sun et al., 2020). The representative protein expression of ADH1C and YTHDC2 was explored in the human protein profiles and is shown in Figure 5B. Nevertheless, ZCRB1 was not found in the HPA website. YTHDC2 possessed the most frequent genetic alterations (3%) among the three genes. Meanwhile, amplification mutation, missense mutation, and deep deletion were the most common alterations among the three genes (Figure 5C). In summary, we further verified the abnormal expression of these three genes in LUAD, and genetic changes may help explain the aberrant expression of these genes to a certain extent.

## Discussion

At the post-transcriptional level, more than 160 kinds of chemical modifications were discovered in various RNAs (Roundtree et al., 2017; Boccaletto et al., 2018). Among these modifications, more and more evidence showed that m^6^A modification had an essential effect on some underlying diseases and prognosis of tumors. Therefore, the identification of m^6^A RNA methylation regulators and m^6^A-related genes in fatal LUAD may offer valuable therapeutic targets to us and clinicians. Doctors usually diagnosed LUAD as advanced, and there was a high death rate with it. Many studies illuminated that the m^6^A process was linked to lung cancer, which made m^6^A RNA methylation regulators and m^6^A-related genes potential biomarkers for clinical practice. According to our research, the classification of m^6^A-related genes in LUAD patients was in association with prognosis. We identified a signature that consisted of one m^6^A RNA methylation regulator (YTHDC2) and two m^6^A-related genes (ZCRB1 and ADH1C) using different statistical and machine learning methods.

Up to now, little is known about the role of YTHDC2 in tumorigenesis. Even less so in LUAD, two studies were found on the role of YTHDC2 in LUAD in recent studies. In a mouse model, low YTHDC2 expression was associated with poor prognosis in LUAD patients, and YTHDC2 improves the prognosis of LUAD patients by inhibiting the independent antioxidant function of SLC7A11 (Ma et al., 2021). In non-small cell lung cancer, a research analyzed a series of publicly available online databases and found that low YTHDC2 expression was associated with lymph node metastasis and poor prognosis (Sun et al., 2020).

ZCRB1 is a zinc finger CCHC-type and RNA binding motif containing 1. A previous study found that it was U12-type splicing playing a pivotal role by RefSeq analysis. However, the function of ZCRB1 was rarely reported in cancer, only in two studies. For example, ZCRB1’s high expression can improve viral replication and transcription (Tan et al., 2012). Through genome-wide analysis of lung adenocarcinoma and healthy subjects, it was found that ZCRB1 may encode viral receptors. COVID-19 has infected plenty of people around the world, and ZCRB1 high expression may impact patients’ prognosis (Cotroneo et al., 2021).

ADH1C is alcohol dehydrogenase 1C (class I). Many reports showed that drinking had an effect on some diseases. High expression of ADH1C was found to protect patients of non-small cell lung cancer (Wang et al., 2018). Using machine learning algorithms, the researchers found that ADH1C could be a prognostic marker (Shen et al., 2019).

For the reversible effect of m^6^A on mRNA expression, we believe that m^6^A-related genes may have different functional patterns and networks when participating in malignant tumors. Thus, m^6^A-related genes may have different expression patterns in LUAD. In previous research, little was known about the interaction of m^6^A-related genes. Moreover, m^6^A RNA methylation regulators (WTAP, RBM15, KIAA1429, YTHDF1, and YTHDF2) were linked with TP53 and highly expressed in TP53 mutant LUAD (Zhuang et al., 2020). However, it is worth nothing that whether the TP53 mutant affects the expression of ZCRB1, ADH1C, and YTHDC2 is still unclear, and more evidence is needed to clarify their mechanism.

## Conclusion

In conclusion, our study systematically analyzed the expression, prognostic value, protein–protein interaction, and potential function of m^6^A RNA methylation regulators and m^6^A-related genes. We found that the expression of m^6^A RNA methylation regulators and m^6^A-related genes was closely related to the clinicopathologial characteristics of LUAD. A three-gene signature was identified that might effectively identify new therapeutic targets or strategies for LUAD. In summary, our study provided important clues for further studies on the role of RNA m^6^A methylation regulators and m^6^A-related genes in LUAD.

## Data Availability Statement

The original contributions presented in the study are included in the article/Supplementary Material, further inquiries can be directed to the corresponding authors.

## Author Contributions

CY, XJ, and WZ: study conception and design. BG, HZ, and CY: manuscript writing. BG, RW, and CY: literature review. All authors: data interpretation, discussion, final editing, and approval of the manuscript in its present form.

## Conflict of Interest

The authors declare that the research was conducted in the absence of any commercial or financial relationships that could be construed as a potential conflict of interest.

## Footnotes

1 http://m6avar.renlab.org/

2 https://www.oncomine.org/resource/main.html

3 https://cistrome.shinyapps.io/timer/

4 http://gepia.cancer-pku.cn/index.html

5 http://www.proteinatlas.org

6 https://www.cbioportal.org/

7 https://string-db.org/

## Supplementary Material

Supplementary Figure 1 Flowchart of the study.

Supplementary Figure 2 The three predictive genes expression levels in LUAD. Data was from the GEPIA database. T, tumor; *N*, normal tissue.

Supplementary Table 1 The list of the 21 m^6^A RNA methylation regulators from publications.

Supplementary Table 2 m^6^A methylation regulators and m^6^A-related genes set of univariate cox regression analysis in the GSE31210 dataset (*P* < 0.01, AUC > 0.6, *n* = 226).

Supplementary Table 3 255 signatures in the GSE31210 dataset (*n* = 226).

## Abbreviations

m^6^A: N6-methyladenosine
LUAD: lung adenocarcinoma
ROC: receiver operating characteristic
GEO: Gene Expression Omnibus
TCGA: The Cancer Genome Atlas
m^1^A: N1-methyladenosine
RSFVH: random survival forest algorithm
lncRNAs: long chain non-coding RNA
OS: overall survival
GO: gene ontology
HR: hazard ratio
CI: confidence interval
KM: Kaplan–Meier.
