# Supplementary figures and images for: Identification of the Signature Associated With m6A RNA Methylation Regulators and m6A-Related Genes and Construction of the Risk Score for Prognostication in Early-Stage Lung Adenocarcinoma
Source: Front Genet. 2021 Jun 11;12:656114. doi: 10.3389/fgene.2021.656114 (PMC8226131; doi:10.3389/fgene.2021.656114)

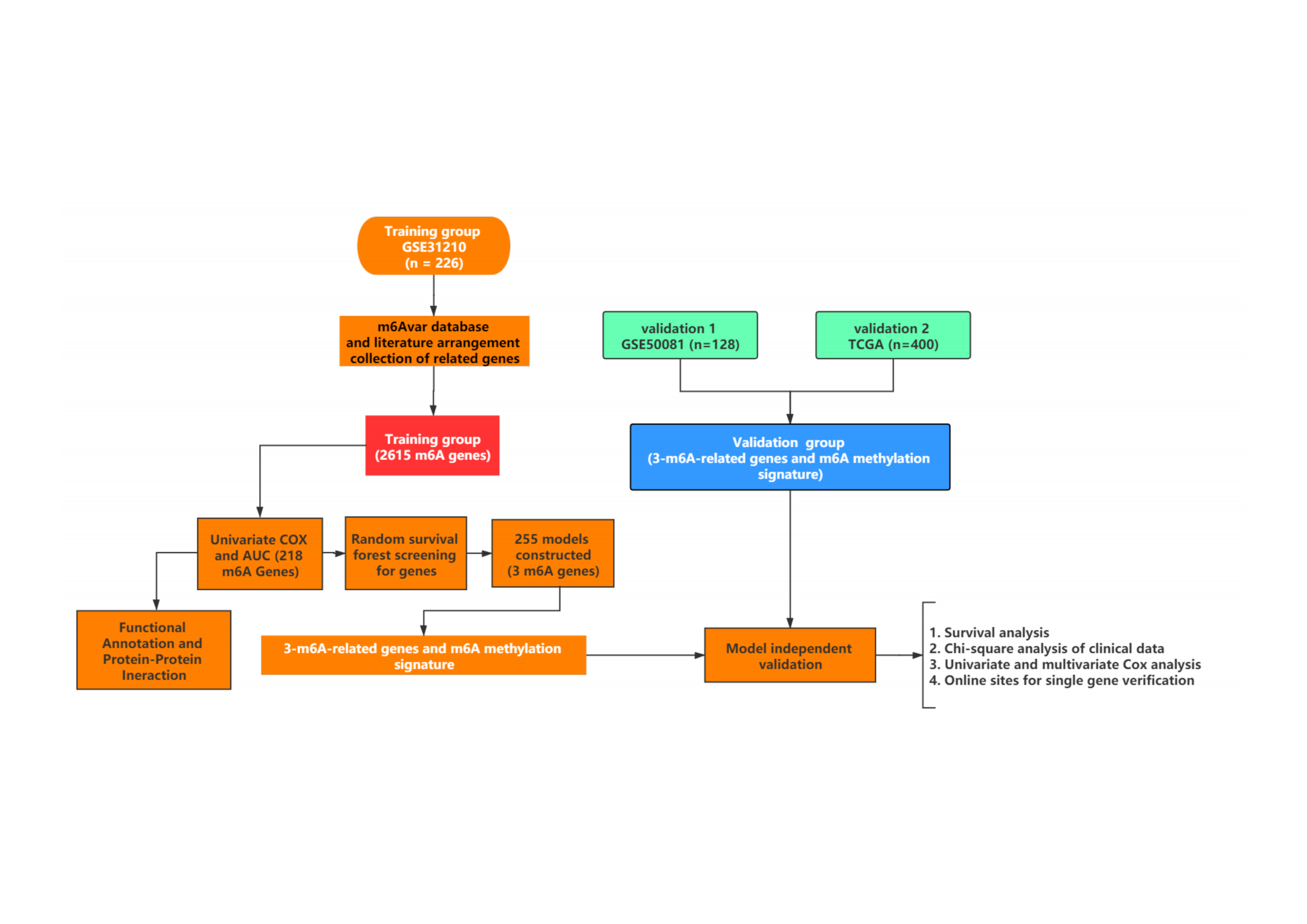

Supplement: Supplementary Figure 1 — Flowchart of the study. [file Image_1.TIF]

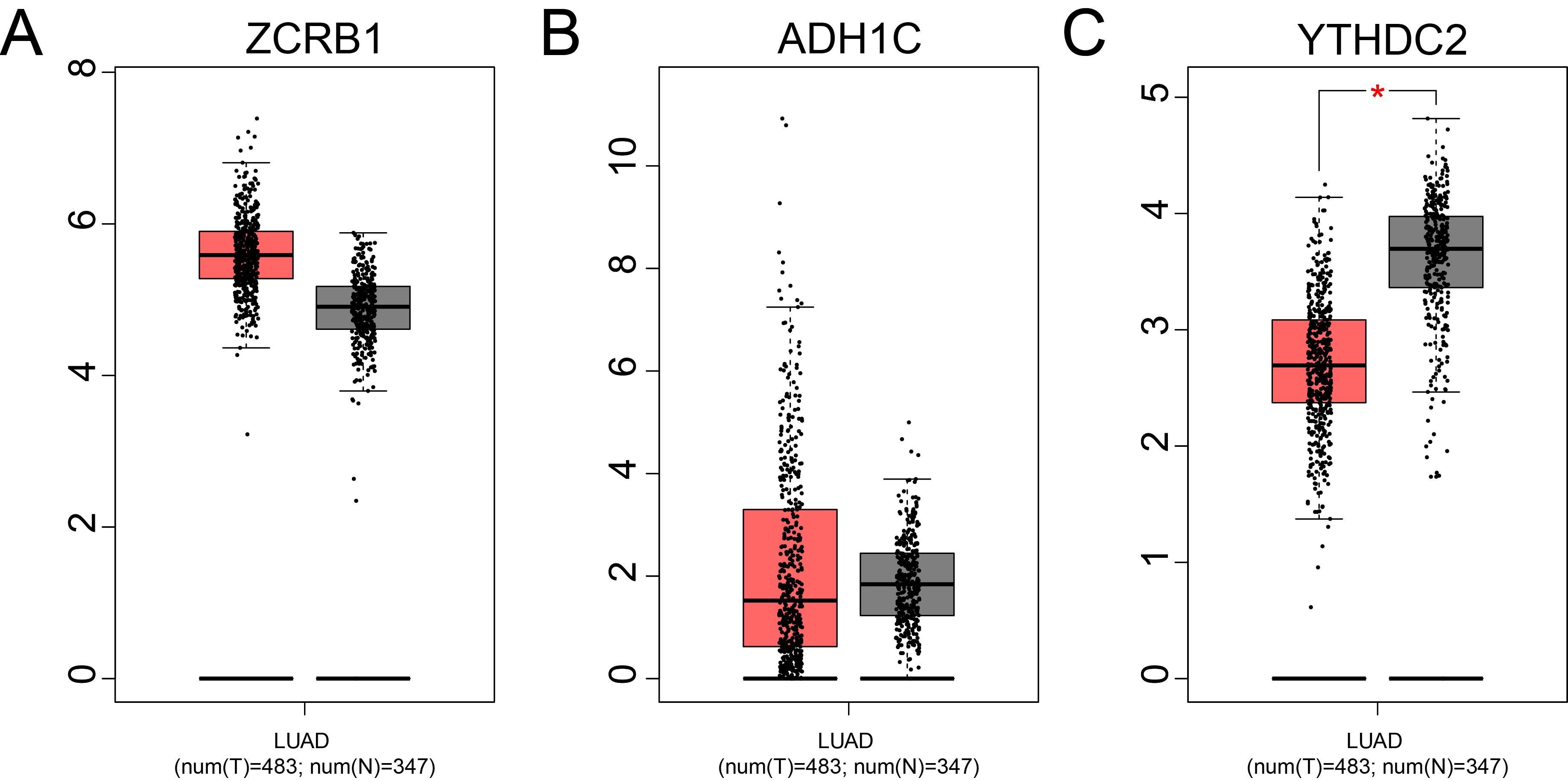

Supplement: Supplementary Figure 2 — The three predictive genes expression levels in LUAD. Data was from the GEPIA database. T, tumor; N, normal tissue. [file Image_2.TIF]
